# TypeLoader2: Automated submission of novel HLA and killer‐cell immunoglobulin‐like receptor alleles in full length

**DOI:** 10.1111/tan.13508

**Published:** 2019-03-25

**Authors:** Bianca Schöne, Markus Fuhrmann, Vineeth Surendranath, Alexander H. Schmidt, Vinzenz Lange, Gerhard Schöfl

**Affiliations:** ^1^ DKMS Life Science Lab Dresden Germany; ^2^ DKMS Tübingen Germany

**Keywords:** annotation, HLA, KIR, novel allele, software, submission

## Abstract

The Immuno Polymorphism Database (IPD) databases provide global, curated repositories for information regarding polymorphisms of genes of the immune system, thereby generating immense value for the research and clinical communities. The advent of high‐throughput genotyping in immunogenetics has led to dramatically growing numbers of heretofore unknown HLA and lately also killer‐cell immunoglobulin‐like receptor (KIR) alleles, which are to be curated and deposited in the IPD‐IMGT/HLA and IPD‐KIR databases, respectively. It is highly desirable that these novel alleles are characterised and submitted in full length, and that known alleles are extended to cover the complete gene sequence.

However, the manual annotation and submission of sequences to European Molecular Biology Laboratory's European Nucleotide Archive and the IPD‐IMGT/HLA and IPD‐KIR databases is time‐consuming and error‐prone. Here, we report the substantial extension of the HLA allele submission tool TypeLoader, which now also supports the annotation and submission of KIR alleles. To enable a more widespread use of this tool, we have made it available as a stand‐alone application that can easily be installed on standard Windows or Linux computers. Furthermore, an internal SQLite database was added to store a wide range of metadata about each allele. This allows TypeLoader2 to be used as a lab's central information platform for the annotation, curation and submission of full‐length HLA and KIR allele sequences. The software is freely available from GitHub (https://github.com/DKMS-LSL/typeloader).

We hope that the increased convenience and scope of TypeLoader2 will foster the submission of more full‐length sequences to the IPD‐IMGT/HLA and IPD‐KIR databases, ultimately promoting the use of full‐length sequencing for genotyping both HLA and KIR.

## 1 INTRODUCTION

The Immuno Polymorphism Database (IPD)^1^ is an invaluable resource for the systematic archival of allele information of the genes of the immune system. The central availability of this curated catalogue of allele sequences enables clinical efforts and research as well as the genotyping of immunogenetic polymorphisms that is indispensable for transplantation of haematopoietic stem cells or for the management of other diseases involving immune reactions. Regularly updating the IPD by way of submitting newly discovered alleles or extending the sequences of partially known alleles is essential to help IPD to reflect the diversity of the immunogenetic landscape. However, at the same time as large‐scale high‐throughput genotyping in immunogenetics has led to rapidly increasing numbers of previously unknown alleles, the manual curation of allele sequences for database submission remains as labour intensive, tedious and error‐prone as ever. Whilst for sequences of the HLA complex, the submission process can be aided by recently introduced software tools,^2^, ^3^ so far there has been no such solution available for killer‐cell immunoglobulin‐like receptor (KIR) alleles.

The recent development of methods for large‐scale allelic‐level KIR genotyping^4^, ^5^ as well as the advent of KIR genotyping in the context of haematopoietic stem cell donor registries^5^ promise a rapid increase in the known variation in KIR (977 catalogued alleles in IPD‐KIR release 2.8.0, November 2018). This prospect makes it prudent to provide a software solution that enables the automated curation and submission of newly discovered KIR allele sequences in a way that is reliably acceptable to IPD‐KIR.

**Figure 1:**
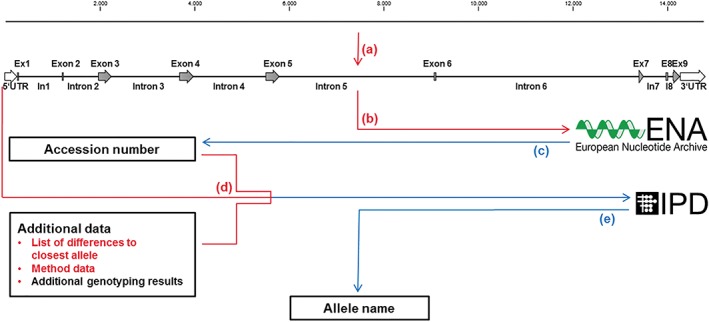
Workflow for submission of novel alleles to the IPD databases. Red arrows and red text indicate steps performed by TypeLoader2, blue arrows denote email communication. (a) The intron‐exon boundaries of the full‐length sequence must be annotated. (b) The annotated sequence must be submitted to a general nucleotide archive like EMBL‐ENA. (c) An accession number will be assigned. (d) The sequence with its accession number and additional metadata can be submitted to the appropriate IPD database. (e) An allele name will be assigned and the allele will be added to the IPD database in question

Allele submission to the IPD‐IMGT/HLA or the IPD‐KIR database is a 2‐step process (Figure 1). In the first of these steps, the sequence along with feature annotations of the allele sequence (exons, introns and untranslated regions (UTRs)) is deposited in a general sequence repository such as the European Molecular Biology Laboratory's European Nucleotide Archive (EMBL‐ENA),^6^ the DNA Data Bank of Japan^7^ or National Center for Biotechnology Information (NCBI) GenBank.^8^ The second step involves transmitting the allele sequence along with its features and the identifier assigned by the sequence repository to IPD. Additionally, IPD requires a delineation of the differences between the novel or extended allele sequence and a closely related existing allele as a coherence check, as well as further metadata about the sample the allele was discovered in and the methods used for sequence characterisation. Each step involves either meticulous entries in a web form or creating a strictly format‐ specific text file. Both of these approaches are cumbersome and highly error‐prone if performed manually, especially when submitting multiple alleles.

In 2016, we developed TypeLoader,^2^ a tool that takes full‐length sequences of novel HLA alleles as input, performs automatic annotations and creates all files necessary for submission. This reduced the hands‐on time for submissions by over 95%. It was implemented as a web application to run on Linux servers and has been used at the DKMS Life Science Lab to successfully submit more than 2400 novel HLA alleles.

To ease and widen the adoption of an automated submission process in other labs, we have implemented a substantially updated version of this submission tool, called TypeLoader2, which can handle both KIR and HLA alleles. In addition, the software has received a completely redesigned graphical user interface (GUI), removing the need for a Linux server backend and converting it from a rather bare‐bones web service to a full‐fledged stand‐alone desktop application. TypeLoader2 runs on Windows as well as Linux and is freely available from GitHub (https://github.com/DKMS-LSL/typeloader). It comes with a convenient installer for Windows as well as setup instructions for Linux. Unlike the previous version, TypeLoader2 features an integrated SQLite database, which can store various metadata about the processed alleles and an intuitive user interface to access these data. Therefore, TypeLoader2 can be used as a central platform to curate, organise and track all the full‐length allele submissions a user is working on.

## 2 METHODS

TypeLoader2 is implemented in Python 3.6^9^ and uses the PyQt5 library^10^ for the GUI and the interactions with the internal SQLite database.^11^ This database file is used to store metadata about all processed alleles, for example, sample IDs, file names, submission status values, accession numbers, cell‐line identifiers and timestamps for process steps. All trackable metadata and the structure of the SQLite database are documented in the user manual that is delivered with the software.

Like its predecessor, TypeLoader2 uses the BioPython^12^ library to parse an input FASTA file containing the full‐length novel allele sequence. The FASTA format is a common and simple text‐based format to represent nucleotide sequences. It can be provided by most sequencing software or easily generated manually using a text editor. An alternative input format for TypeLoader2 are the XML files exported from NGSengine,^13^ a software for the high‐resolution identification of HLA and KIR alleles using next‐ and third‐generation sequencing. These files are parsed using the xmltodict Python library.^14^

The reference databases hla.dat and KIR.dat are downloaded from https://github.com/ANHIG/IMGTHLA/raw/Latest/hla.dat and https://github.com/ANHIG/IPDKIR/raw/Latest/KIR.dat, respectively. TypeLoader2 uses a custom EMBL‐ENA parser to retrieve all full‐length reference allele sequences and their gene feature annotations from these files.

The processes of gene feature annotation and submission file generation have been described previously.^2^ Briefly, the reference allele closest to the novel allele in sequence space is determined using BLAST.^15^ Annotations of exons, introns and UTRs for the novel allele are then derived from the annotations of this reference allele. Subsequently, the BLAST output is parsed using the BLAST parser from the BioPython library to elucidate differences between the novel allele and the reference allele.

The direct submission of projects and alleles to EMBL‐ENA is handled via a Representational State Transfer (REST)^16^ interface using the PycURL library.^17^

The TypeLoader2 executable for Windows is created using the cx_Freeze library for Python,^18^ and the corresponding installer is generated using the nullsoft scriptable install system (NSIS).^19^

TypeLoader2 stores all user account data (user names and passwords) locally in a hashed text file. All generated data and files are stored locally under a path specified by the user during setup and only shared with the sequence repositories at the user's request, so that users have complete control over all their data at all times.

## 3 RESULTS

### 3.1 Using TypeLoader2

#### 3.1.1 Setup

Like the previous version of the software, TypeLoader2 uses EMBL‐ENA as its general nucleotide archive of choice. Before being able to submit alleles to the EMBL‐ENA and the IPD databases, users will have to register with both databases as submitters and configure their TypeLoader2 user account with the submitter IDs they receive. This configuration is ideally performed during the installation process of TypeLoader2, but can also be performed at a later point in time.

For Windows, a TypeLoader2 installer can be downloaded that includes all dependencies and asks the user for all data necessary to configure the software. For Linux, the dependencies must be installed separately and the configuration files need to be created manually, as detailed in the user manual.

During setup, the user is prompted to specify a data path under which TypeLoader2 will store all generated files. This can be a folder on the local computer or a network path, allowing for centralised data storage in a multi‐user lab.

After setup, the user is prompted to create a local user account, which will be used to store the data of all alleles handled by a single user. This allows multiple users within one laboratory to use the software on multiple computers simultaneously, each under a different user account. If no simultaneous usage is required, creating one user account for the whole laboratory may be easiest.

Unlike its predecessor, TypeLoader2 communicates with EMBL‐ENA directly, via ENA's REST representational state transfer application programming interface (API).^20^ Therefore, we strongly recommend creating a test user account in TypeLoader2 first, which will communicate with EMBL‐ENA's test server instead of their productive server. All test submissions are automatically deleted by EMBL‐ENA after 24 hours, providing a straightforward mechanism to become familiar with the software and to verify that the connection is set up correctly and everything works as expected.

#### 3.1.2 Creating a project

Before submitting individual alleles to EMBL‐ENA, the user has to create an EMBL‐ENA project, which may correspond to a pool of samples or a study. To remove the need to use EMBL‐ENA's web form for this step, TypeLoader2 offers a dialogue box which prompts the user for the required information, submits it directly to EMBL‐ENA's REST server, and automatically receives a project submission number and project accession number from EMBL‐ENA. This information is then used by the software to create a TypeLoader2 project with a corresponding directory in the user's data space, where all files related to this project will be stored.

#### 3.1.3 Adding an allele

Once a project has been created, an allele sequence can be added by uploading an individual FASTA or XML file containing the full‐length sequence and by providing a corresponding sample ID. Sequences that do not contain the full gene including both UTRs are currently rejected, although a future version will allow adding genomic sequences with partially known UTRs, as well. TypeLoader2 then searches the reference database for the reference allele from the IPD‐IMGT/HLA or IPD‐KIR database, as appropriate, that is closest in sequence space to the uploaded sequence of the allele being processed. This provides the exon‐intron boundaries of the novel sequence. The annotated sequence is subsequently formatted for submission to EMBL‐ENA and stored in the project directory. The gene annotation process has been described previously in more detail.^2^

Unlike the previous version, TypeLoader2 also offers a bulk upload option for FASTA files, reducing hands‐on time for experienced users further by de‐coupling the manual upload of files and IDs from the automated gene annotation by TypeLoader2. Furthermore, the new software version automatically detects null alleles generated by frameshift mutations or preliminary stop codons, displays this information in the GUI and formats the created submission files accordingly.

#### 3.1.4 Submitting alleles to EMBL‐ENA

To submit alleles to EMBL‐ENA, a user chooses a project and selects the alleles ready for submission. TypeLoader2 proceeds to generate the necessary concatenated EMBL flatfile and the corresponding XML file required by EMBL‐ENA, and transfers these files directly to EMBL‐ENA's REST server. Subsequently, TypeLoader2 parses the EMBL server response and notifies the user of the status of the submission. Rejections are qualified with the identifiers of the rejected alleles and the reasons for rejection. The internal processing status for each submitted allele is then updated accordingly.

#### 3.1.5 Submitting to IPD

After a successful submission to EMBL‐ENA, the nucleotide archive will assign unique accession numbers to all submitted alleles and send these to the submitter as an email attachment. To submit the alleles to IPD‐KIR or IPD‐IMGT/HLA, the user chooses a project and provides this email attachment along with a comma separated value (CSV) file containing the genotyping results for all other HLA and/or KIR loci of the samples to be submitted. IPD currently requires at a minimum the genotypes for HLA‐A, HLA‐B and HLA‐DRB1 as well as the second allele of the submitted locus, but strongly encourages the inclusion of all other available genotyping information of the same sample. TypeLoader2 uses the information contained in these files, the already stored submission data, as well as information on the sequencing methods used. The latter is entered via the GUI, which TypeLoader2 explicitly demands during first use of this functionality and stored in configuration files. From these data, TypeLoader2 creates all files necessary for IPD submission. Subsequently, the user can download these files as a .zip archive and send them to IPD via their assigned channel (see Section 3.1.6).

Once IPD has approved the submitted novel alleles and assigned allele names, the submitter receives an email response containing the IPD accession number and the official allele name for each submitted allele. Currently, this information can be stored in TypeLoader2 only by entering it manually via the user interface. A future version of TypeLoader2 will, however, be able to parse these email attachments automatically and extract the relevant information.

#### 3.1.6 Registering with IPD as a bulk submitter

To ensure that IPD's high quality criteria are met by all submitters, new users will have to contact the IPD submissions team before they can directly submit sequence files created with TypeLoader2. Instructions for this one‐time process, which includes the submission of several confirmatory as well as novel alleles, are given in the user manual. Once IPD is satisfied that all requirements are fulfilled, they will provide the user with directions on how to submit further TypeLoader2 generated files directly, either by email or a shared drive, depending on the expected volume and frequency of allele submissions.

Users who only wish to submit a low number of alleles can skip this process by using the files generated by TypeLoader2 as a source to copy and paste the necessary parts into IPD's web submission form, which is the recommended way to submit a first allele. Detailed instructions on how to do this are given in the TypeLoader2 manual.

### 3.2 The Graphical User Interface of TypeLoader2

#### 3.2.1 General structure and navigation

**Figure 2:**
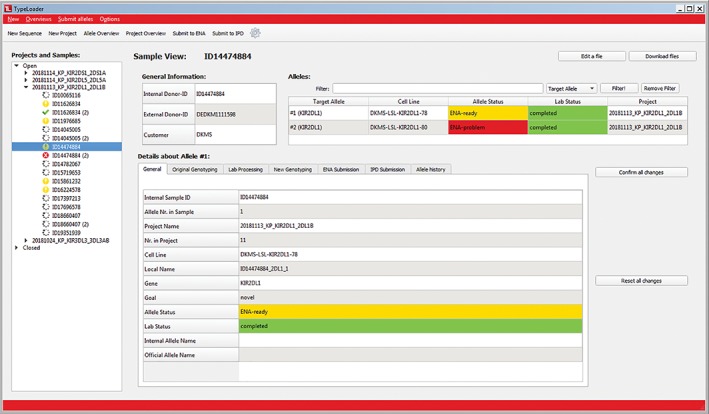
The graphical user interface (GUI) of TypeLoader2, displaying in the main panel the detailed “Sample View” for a single sample containing two alleles. This view presents information about the alleles of a sample selected for submission (“target alleles”), including general information about the sample (top left), a list of the target alleles (top right), as well as a section displaying various metadata about a single allele (bottom). The data in this view can be edited except where protected for consistency reasons. All changes must be confirmed or reset before leaving the view to prevent accidental data changes. The buttons (top right) allow accessing, downloading, or editing of files associated with the sample. Other detailed views about items of interest (projects or other alleles) can be accessed through the navigation area (left panel)

TypeLoader2 has been designed to offer the user convenient access to all their full‐length alleles meant for submission. These can be novel allele sequences, sequences submitted to extend partially described alleles, or previously described alleles submitted for confirmatory purposes. The GUI is divided into three main panels (Figure 2): a menu area on the top, a large main area underneath and a navigation area for convenient access to any item of interest (either a project or an allele) on the left.

Each target allele receives an “allele status” representing its overall state within the workflow, as well as a “lab status” representing its status within the “wet lab” workflow of allele characterisation. Values for both are structured into different categories, each associated with a colour and an icon: finished (green check mark), pending response from ENA‐EMBL or IPD (grey circle of dots), to‐do (yellow exclamation mark) or error (red x). The alleles in the navigation area are marked with icons corresponding to their allele status, so the user can easily see which alleles currently need user interaction (the yellow and red alleles).

#### 3.2.2 Main views

After logging into TypeLoader2, the main area displays a concise view of all projects owned by the user (Supplementary Figure 1, Supporting Information). This “Project Overview” provides easy access to the user's projects. Likewise, the “Allele Overview” (Supplementary Figure 2) can be used to find and access any allele of interest. Both views can be filtered and sorted by all columns and are non‐editable. A detailed view of any item of interest can be opened from these main views or using the navigation area.

#### 3.2.3 Detailed views

Any detailed view in the user interface provides the ability to access or edit data corresponding to one item of interest, that is, a project (“Project View,” Supplementary Figure 3), or a sample with its alleles (“Sample View,” Figure 2). These views show all metadata about the item of interest and can be used to access all files associated with this item.

The “Project View” can also be used to toggle the status of a project between “Open” and “Closed.” “Closed” projects are hidden by default in the “Project Overview” (Supplementary Figure 1), allowing easier display of and access to ongoing projects. In the navigation area, hidden projects appear in the “Closed” section, which is displayed below all open projects and collapsed by default.

The “Sample View” can be used to access and edit all metadata and files associated with any target allele contained in a sample. For user convenience, the information is presented in tabs. Any shifting of interface focus subsequent to an edit action requires user confirmation to prevent accidental changes by the user.

#### 3.2.4 Dialogue boxes

**Figure 3:**
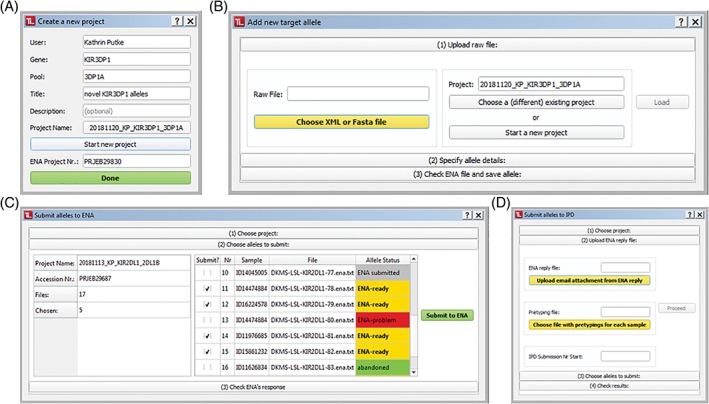
Dialog boxes. Adding and submitting data through TypeLoader2 is performed via popup dialogs, which guide the user through all necessary steps. (A) New project dialog. (B) New allele dialog. (C) EMBL‐ENA submission dialog. (D) IPD submission dialog

Adding new projects or alleles as well as allele submission is handled via popup dialogues (Figure 3), which can be accessed using the menu or tool bar on the top of the GUI. Every dialogue interaction guides the user with appropriately coloured interface elements, with yellow indicating required actions and green indicating readiness to proceed. Where reasonable, subsequent steps of the workflow translate into separate interface sections that expand and collapse corresponding to the user's progress through the workflow, reducing visual overload and confusion.

### 3.3 Novel alleles submitted with TypeLoader

**Figure 4:**
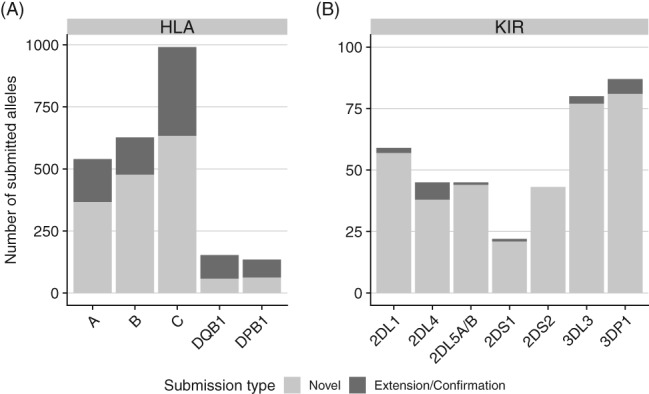
Number of alleles submitted by DKMS Life Science Lab via TypeLoader (as of November 2018) to IPD‐IMGT/HLA (A) and IPD‐KIR (B). This includes alleles submitted by both versions of TypeLoader

At the time of writing, the DKMS Life Science Lab has used TypeLoader (in both versions) to successfully submit 2827 full‐length alleles to IPD, including 381 KIR alleles (Figure 4). These figures show TypeLoader's capability to handle large throughput with minimal user interaction.

## 4 DISCUSSION

With the increasing availability of next generation genotyping (NGS)‐based genotyping methods for KIR, there is a growing number of laboratories that sequence substantial numbers of KIR alleles in the context of basic research as well as first efforts to establish KIR genotyping in the context of high‐throughput stem‐cell donor registry typing.^4^, ^5^, ^21^ We expect that these efforts will lead to the discovery of a large number of novel KIR alleles,^5^ as was the case with the HLA genes in the previous years.^22^ However, in contrast to HLA, there is currently no software available to support the submission of full‐length KIR alleles.

Because sequence repositories demand precise encoding and formatting of all submitted data, manually preparing novel allele data for submission is highly time‐consuming and error‐prone. Consequently, without automation of substantial parts of the annotation and submission process, laboratories will be discouraged from submitting newly discovered KIR alleles to ENA and IPD.

Therefore, we have substantially updated the HLA submission software TypeLoader^2^ to handle KIR as well as HLA, thus, to our knowledge, providing the first tool for automated KIR allele submission. Additionally, we have significantly reduced the remaining hands‐on time for the submission process by implementing bulk upload of sequence data and by integrating TypeLoader2 directly with the EMBL‐ENA REST API, thereby enabling the software to handle the complete communication with EMBL‐ENA autonomously.

At the same time, TypeLoader2 features a substantially improved user interface, enabling swift and intuitive processing of target alleles by the user. With the integrated SQLite database, it provides a convenient centralised platform for managing all novel alleles characterised in a laboratory. This includes handling all relevant metadata of alleles, which allows the software to serve as a minimal laboratory information management system (LIMS) for full‐length sequences meant for submission.

To simplify the usage of TypeLoader for other labs, we have developed TypeLoader2 as a stand‐alone desktop application, which, unlike its predecessor, requires no Linux server backend. For Windows, it comes with a convenient installer including all necessary dependencies. For Linux, straightforward setup instructions are given in the user manual. At the same time, the full source code is available, making the software easy to adapt to a lab's individual needs and workflows.

An alternative HLA submission tool, Saddlebags,^3^ also features integration with the EMBL‐ENA REST API, but this software does not yet provide submission files that are accepted by IPD. Moreover, it cannot handle KIR and is tailored towards individual allele submission, providing none of the LIMS‐like functionality of TypeLoader2.

We hope that the increased usability and scope of TypeLoader2 will encourage other labs performing full‐length HLA/KIR sequencing to submit novel or so far only partially known alleles to the IPD‐IMGT/HLA and IPD‐KIR databases. This software should enable researchers working on novel alleles to spend more of their time on characterising their sequences and less on the submission process, ultimately providing the HLA and KIR communities with more comprehensive IPD databases.

## CONFLICT OF INTEREST

The authors have declared no conflicting interests.

## Supporting information


**Figure S1.**
Supplementary Figure 1. The “Project Overview” is the main entry point of the graphical user interface of TypeLoader2. All allele submission projects are listed in the main panel, which can be sorted or filtered by each column. Closed projects, which are hidden by default, can be included using the “Show closed projects” button (top left in the main panel). Individual projects can be accessed using the right‐click menu of the main panel or the navigation area (left panel).

**Figure S2.**
Supplementary Figure 2. The “Allele Overview” presents all alleles owned by a user, including all associated metadata. Like in the “Project Overview” (Supplementary Figure 1), the table can be sorted and filtered by all columns but is non‐editable. It can be used to find a specific allele of interest and open it in the corresponding “Sample View” (Figure 2).

**Figure S3.**
Supplementary Figure 3. The “Project View” for an individual submission project presents information about a single project, including statistical data (top left), project creation data (bottom left), and a list of all alleles contained in the project (right). This view can be used to toggle the project status between “Open” and “Closed”, and to access associated files. Single allele data can be accessed from the allele list via right click, or by using the navigation area.
