# Supplementary material for: TypeLoader2: Automated submission of novel HLA and killer‐cell immunoglobulin‐like receptor alleles in full length
Source: HLA. 2019 Mar 25;93(4):195–202. doi: 10.1111/tan.13508 (PMC6594033; doi:10.1111/tan.13508)
Supplement: Supplementary file 1 — Figure S1. Supplementary Figure 1. The “Project Overview” is the main entry point of the graphical user interface of TypeLoader2. All allele submission projects are listed in the main panel, which can be sorted or filtered by each column. Closed projects, which are hidden by default, can be included using the “Show closed projects” button (top left in the main panel). Individual projects can be accessed using the right‐click menu of the main panel or the navigation area (left panel). [file TAN-93-195-s001.pdf]

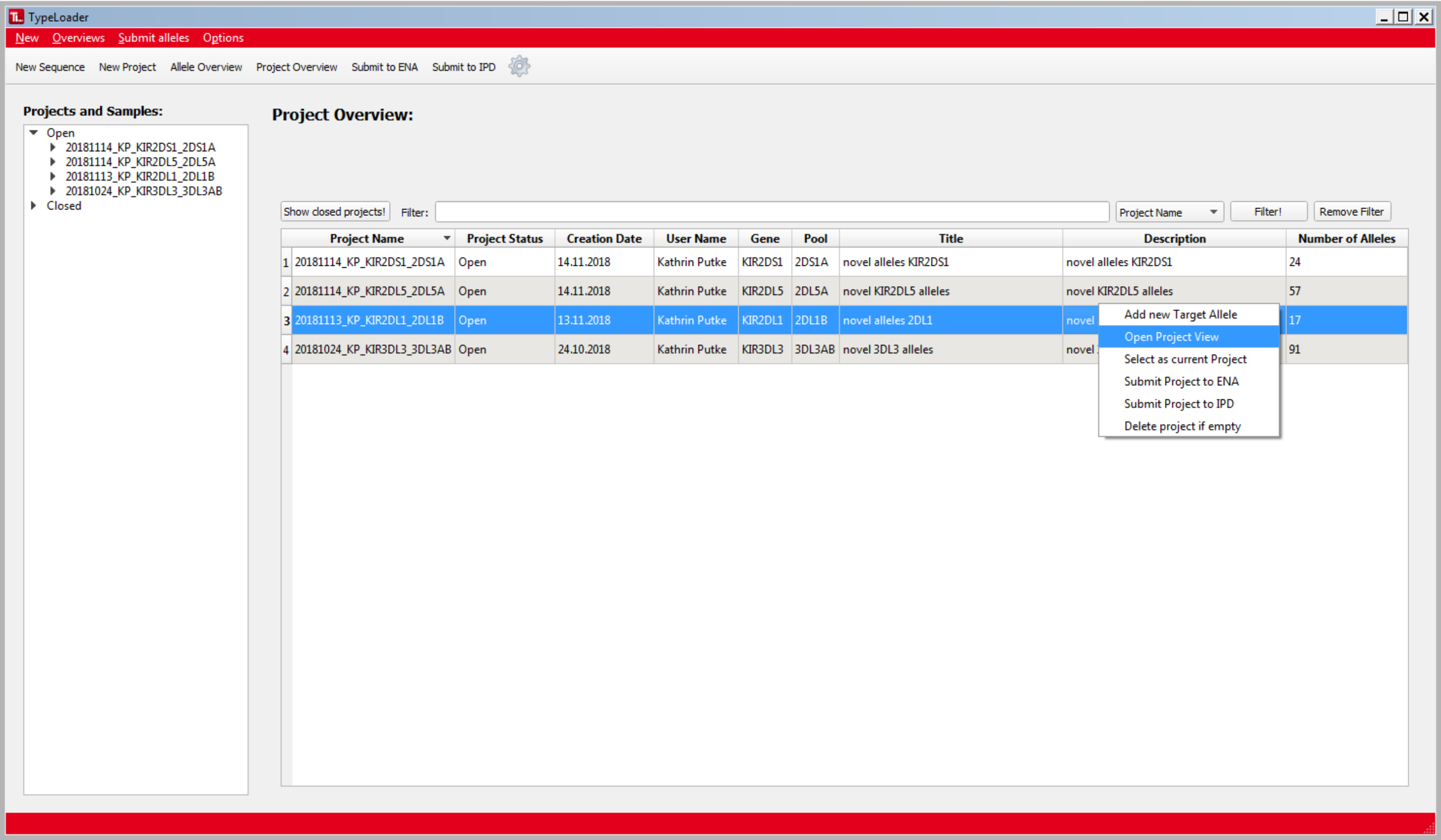
TypeLoader
New Overviews Submit alleles Options
New Sequence New Project Allele Overview Project Overview Submit to ENA Submit to IPD
Projects and Samples:
Open
20181114_KP_KIR2DS1_2DS1A
20181114_KP_KIR2DL5_2DL5A
20181113_KP_KIR2DL1_2DL1B
20181024_KP_KIR3DL3_3DL3AB
Closed
Project Overview:
Show closed projects! Filter: Project Name Filter! Remove Filter
Project Name | Project Status | Creation Date | User Name | Gene | Pool | Title | Description | Number of Alleles
1 20181114_KP_KIR2DS1_2DS1A | Open | 14.11.2018 | Kathrin Putke | KIR2DS1 | 2DS1A | novel alleles KIR2DS1 | novel alleles KIR2DS1 | 24
2 20181114_KP_KIR2DL5_2DL5A | Open | 14.11.2018 | Kathrin Putke | KIR2DL5 | 2DL5A | novel KIR2DL5 alleles | novel KIR2DL5 alleles | 57
3 20181113_KP_KIR2DL1_2DL1B | Open | 13.11.2018 | Kathrin Putke | KIR2DL1 | 2DL1B | novel alleles 2DL1 | novel | 17
4 20181024_KP_KIR3DL3_3DL3AB | Open | 24.10.2018 | Kathrin Putke | KIR3DL3 | 3DL3AB | novel 3DL3 alleles | novel | 91
Add new Target Allele
Open Project View
Select as current Project
Submit Project to ENA
Submit Project to IPD
Delete project if empty
