# Supplementary material for: TypeLoader2: Automated submission of novel HLA and killer‐cell immunoglobulin‐like receptor alleles in full length
Source: HLA. 2019 Mar 25;93(4):195–202. doi: 10.1111/tan.13508 (PMC6594033; doi:10.1111/tan.13508)
Supplement: Supplementary file 2 — Figure S2. Supplementary Figure 2. The “Allele Overview” presents all alleles owned by a user, including all associated metadata. Like in the “Project Overview” (Supplementary Figure 1), the table can be sorted and filtered by all columns but is non‐editable. It can be used to find a specific allele of interest and open it in the corresponding “Sample View” (Figure 2). [file TAN-93-195-s002.pdf]

TypeLoader

New Overviews Submit alleles Options

New Sequence New Project Allele Overview Project Overview Submit to ENA Submit to IPD

Projects and Samples:

- Open
  - 20181114_KP_KIR2DS1_2DS1A
  - 20181114_KP_KIR2DL5_2DL5A
  - 20181113_KP_KIR2DL1_2DL1B
  - 20181024_KP_KIR3DL3_3DL3AB
- Closed

Allele Overview:

Filter: Internal Sample ID Filter! Remove Filter

	Internal Sample ID	Allele Nr. in Sample	Project Name	Nr. in Project	Cell Line	Local Name	Gene	Goal	Allele Status	Original Allele #1	Ori
1	ID14362529	1	20181114_KP_KIR2DS1_2DS1A	1	DKMS-LSL-KIR2DS1-1	ID14362529_2DS1_1	KIR2DS1	novel	IPD submitted		
2	ID18278598	1	20181114_KP_KIR2DS1_2DS1A	2	DKMS-LSL-KIR2DS1-2	ID18278598_2DS1_1	KIR2DS1	novel	IPD submitted		
3	ID10389037	1	20181114_KP_KIR2DS1_2DS1A	3	DKMS-LSL-KIR2DS1-3	ID10389037_2DS1_1	KIR2DS1	novel	IPD submitted		
4	ID15918912	1	20181114_KP_KIR2DS1_2DS1A	4	DKMS-LSL-KIR2DS1-4	ID15918912_2DS1_1	KIR2DS1	extend	IPD submitted		
5	ID12861362	1	20181114_KP_KIR2DS1_2DS1A	5	DKMS-LSL-KIR2DS1-5	ID12861362_2DS1_1	KIR2DS1	extend	IPD submitted		
6	ID14582611	1	20181114_KP_KIR2DS1_2DS1A	6	DKMS-LSL-KIR2DS1-6	ID14582611_2DS1_1	KIR2DS1	novel	IPD submitted		
7	ID14362529	2	20181114_KP_KIR2DS1_2DS1A	7	DKMS-LSL-KIR2DS1-7	ID14362529_2DS1_2	KIR2DS1	novel	abandoned		
8	ID10510365	1	20181114_KP_KIR2DS1_2DS1A	8	DKMS-LSL-KIR2DS1-8	ID10510365_2DS1_1	KIR2DS1	novel	IPD submitted		
9	ID18610090	1	20181114_KP_KIR2DS1_2DS1A	9	DKMS-LSL-KIR2DS1-9	ID18610090_2DS1_1	KIR2DS1	confirm	IPD submitted		
10	ID19890222	1	20181114_KP_KIR2DS1_2DS1A	10	DKMS-LSL-KIR2DS1-10	ID19890222_2DS1_1	KIR2DS1	novel	ENA-problem		
11	ID16519526	1	20181114_KP_KIR2DS1_2DS1A	11	DKMS-LSL-KIR2DS1-11	ID16519526_2DS1_1	KIR2DS1	novel	IPD submitted		
12	ID15506023	1	20181114_KP_KIR2DS1_2DS1A	12	DKMS-LSL-KIR2DS1-12	ID15506023_2DS1_1	KIR2DS1	novel	IPD submitted		
13	ID18720846	1	20181114_KP_KIR2DS1_2DS1A	13	DKMS-LSL-KIR2DS1-13	ID18720846_2DS1_1	KIR2DS1	novel	IPD submitted		
14	ID16092957	1	20181114_KP_KIR2DS1_2DS1A	14	DKMS-LSL-KIR2DS1-14	ID16092957_2DS1_1	KIR2DS1	novel	IPD submitted		
15	ID13303503	1	20181114_KP_KIR2DS1_2DS1A	15	DKMS-LSL-KIR2DS1-15	ID13303503_2DS1_1	KIR2DS1	confirm	ENA-ready		
16	ID15304132	1	20181114_KP_KIR2DS1_2DS1A	16	DKMS-LSL-KIR2DS1-16	ID15304132_2DS1_1	KIR2DS1	novel	ENA-ready		
17	ID12165299	1	20181114_KP_KIR2DS1_2DS1A	17	DKMS-LSL-KIR2DS1-17	ID12165299_2DS1_1	KIR2DS1	novel	ENA submitted		
18	ID13659720	1	20181114_KP_KIR2DS1_2DS1A	18	DKMS-LSL-KIR2DS1-18	ID13659720_2DS1_1	KIR2DS1	novel	ENA submitted		
