# Supplementary material for: TypeLoader2: Automated submission of novel HLA and killer‐cell immunoglobulin‐like receptor alleles in full length
Source: HLA. 2019 Mar 25;93(4):195–202. doi: 10.1111/tan.13508 (PMC6594033; doi:10.1111/tan.13508)
Supplement: Supplementary file 3 — Figure S3. Supplementary Figure 3. The “Project View” for an individual submission project presents information about a single project, including statistical data (top left), project creation data (bottom left), and a list of all alleles contained in the project (right). This view can be used to toggle the project status between “Open” and “Closed”, and to access associated files. Single allele data can be accessed from the allele list via right click, or by using the navigation area. [file TAN-93-195-s003.pdf]

TypeLoader

New Overviews Submit alleles Options

New Sequence New Project Allele Overview Project Overview Submit to ENA Submit to IPD

Projects and Samples:

- Open
  - 20181114_KP_KIR2DS1_2DS1A
  - 20181114_KP_KIR2DL5_2DL5A
  - 20181113_KP_KIR2DL1_2DL1B
    - ID10065116
    - ID11626834
    - ID11626834 (2)
    - ID11976685
    - ID14045005
    - ID14045005 (2)
    - ID14474884
    - ID14474884 (2)
    - ID14782067
    - ID15719653
    - ID15861232
    - ID16224578
    - ID17397213
    - ID17696578
    - ID18660407
    - ID18660407 (2)
    - ID19351939
  - 20181024_KP_KIR3DL3_3DL3AB
- Closed

Project View: 20181113_KP_KIR2DL1_2DL1B

Statistics:

Number of alleles	17
Closed alleles	1
Submitted to ENA	10
Submitted to IPD	0
Accepted by IPD	0
Abandoned	1

General Information:

Project Status	Open
Created on	13.11.2018
Created by	Kathrin Putke
Gene	KIR2DL1
Pool	2DL1B
Title	novel alleles 2DL1
Description	novel alleles 2DL1
ENA Project ID	PRJEB29687
ENA Project Submission ID	ERA1654530

Close Project Download files Edit a file

Alleles:

Filter: Target Allele Filter! Remove Filter

Nr	Target Allele	Cell Line	Allele Status	Lab Status
1	ID15719653 #1 (KIR2DL1)	DKMS-LSL-KIR2DL1-27	ENA submitted	completed
2	ID14045005 #1 (KIR2DL1)	DKMS-LSL-KIR2DL1-69	ENA submitted	completed
3	ID14782067 #1 (KIR2DL1)	DKMS-LSL-KIR2DL1-70	ENA submitted	completed
4	ID10065116 #1 (KIR2DL1)	DKMS-LSL-KIR2DL1-71	ENA submitted	completed
5	ID17696578 #1 (KIR2DL1)	DKMS-LSL-KIR2DL1-72	ENA submitted	completed
6	ID18660407 #1 (KIR2DL1)	DKMS-LSL-KIR2DL1-73	ENA submitted	completed
7	ID17397213 #1 (KIR2DL1)	DKMS-LSL-KIR2DL1-74	ENA submitted	completed
8	ID18660407 #2 (KIR2DL1)	DKMS-LSL-KIR2DL1-75	ENA submitted	completed
9	ID19351939 #1 (KIR2DL1)	DKMS-LSL-KIR2DL1-76	ENA submitted	completed
10	ID14045005 #2 (KIR2DL1)	DKMS-LSL-KIR2DL1-77	ENA submitted	completed
11	ID14474884 #1 (KIR2DL1)	DKMS-LSL-KIR2DL1-78	ENA-ready	completed
12	ID16224578 #1 (KIR2DL1)	DKMS-LSL-KIR2DL1-79	ENA-ready	completed
13	ID14474884 #2 (KIR2DL1)	DKMS-LSL-KIR2DL1-80	ENA-problem	completed
14	ID11976685 #1 (KIR2DL1)	DKMS-LSL-KIR2DL1-81	ENA-ready	completed
15	ID15861232 #1 (KIR2DL1)	DKMS-LSL-KIR2DL1-82	ENA-ready	completed
16	ID11626834 #1 (KIR2DL1)	DKMS-LSL-KIR2DL1-83	ENA-ready	completed
17	ID11626834 #2 (KIR2DL1)	DKMS-LSL-KIR2DL1-84	abandoned	completed
